# Harmine Targets Peroxiredoxin 6 to Enhance Macrophage Immunity Against *Pseudomonas plecoglossicida* in Ayu (*Plecoglossus altivelis*)

**DOI:** 10.3390/antiox15040477

**Published:** 2026-04-11

**Authors:** Yan-Jun Liu, Xiang Li, Yi-Fang Jiang, Ran Wang, Jing Yu, Zhi-Guo Liu, Jia-Feng Cao, Guan-Jun Yang, Jiong Chen

**Affiliations:** 1 State Key Laboratory for Quality and Safety of Agro-Products, School of Marine Sciences, Ningbo University, Ningbo 315211, China; 2 Key Laboratory of Aquacultural Biotechnology of Ministry of Education, Ningbo University, Ningbo 315211, China

**Keywords:** *Plecoglossus altivelis*, *Pseudomonas plecoglossicida*, peroxiredoxin 6, monocytes/macrophages, host-directed immunomodulation, aquaculture, herbal medicine

## Abstract

*Pseudomonas plecoglossicida* causes bacterial hemorrhagic ascites in ayu (*Plecoglossus altivelis*), a lethal disease characterized by abdominal distension with hemorrhagic ascites, multifocal organ hemorrhages, and histopathologically evident hepatocellular necrosis and inflammatory infiltration. The lack of effective treatments exacerbates mass mortalities, posing a significant threat to aquaculture. Given the severe pathogenesis of *P. plecoglossicida* infection—which involves bacterial colonization, tissue necrosis, and host immune dysregulation—effective therapeutic strategies are urgently needed. Through a screen of traditional Chinese medicine monomers, we identified harmine, an indole alkaloid derived from *Peganum harmala* seeds, as a potent agent against this pathogen. In vivo, harmine exhibited direct bactericidal activity by disrupting membrane integrity, as evidenced by increasing membrane permeability, and inhibiting biofilm formation. In an ayu infection model, harmine significantly increased host survival, reduced tissue bacterial load, and enhanced innate immunity by augmenting monocyte/macrophage phagocytosis and bactericidal capacity while suppressing pro-inflammatory cytokine release and apoptosis. Mechanistically, the Drug Affinity Responsive Target Stability assay was used to identify the molecular target of harmine, followed by functional validation through *PRDX6*−knockdown experiments. Harmine exhibited direct bactericidal activity by disrupting membrane integrity and inhibiting biofilm formation. In the ayu infection model, harmine significantly increased host survival, reduced tissue bacteria1 load, and enhanced innate immunity by augmenting monocyte/macrophage system and bactericidal capacity while suppressing pro-inflammatory cytokine release and apoptosis, the latter likely through modulation of PRDX6−mediated oxidative stress and downstream caspase signaling. Mechanistically, DARTS revealed that harmine binds to peroxiredoxin 6 (PRDX6), a multifunctional enzyme possessing peroxidase, phospholipase A_2_, and lysophosphatidylcholine acyltransferase activities. This binding liberates TNF receptor-associated factor 6 (TRAF6), facilitating its mitochondrial translocation and association with the ECSIT signaling integrator complex, thereby amplifying mitochondrial reactive oxygen species (mROS) production and potentiating macrophage-mediated bacterial killing. These findings establish harmine as a promising therapeutic candidate for controlling *P. plecoglossicida* infections and underscore the value of host-directed immunomodulation derived from natural products in aquaculture medicine.

## 1. Introduction

*Plecoglossus altivelis* (Ayu) is an economically important fish species prized for its high-quality meat [1]. However, the intensification of aquaculture has exacerbated environmental stress and disease outbreaks, threatening industry sustainability [1]. *Pseudomonas plecoglossicida* is a rod−shaped, flagellated Gram-negative bacterium and the high mortality causative agent of bacterial hemorrhagic ascites (BHA) [2] and visceral white spot disease in commercially vital marine and freshwater aquaculture species [3]. BHA, characterized by visceral hemorrhage, ascites, and multifocal systemic necrosis, causes high mortality in ayu and affects a broad range of farmed fish, including *Larimichthys crocea* and *Epinephelus coioides* [3,4,5]. The lack of effective therapeutics forces reliance on cumbersome preventive measures, underscoring an urgent need for targeted control strategies [1]. Critically, recent clinical reports have raised a public health concern that fundamentally alters this pathogen’s risk profile: the isolation of *P. plecoglossicida* from human cerebrospinal fluid and the lungs of children with pneumonia [6,7]. The former cases, occurring in healthy adults engaged in water-based activities such as small-scale mining, confirm significant zoonotic potential [6], and later case was found a child with infectious pneumonia [7]. This species jumps from aquatic ecosystems to human hosts—particularly causing meningitis—and thus transforms *P. plecoglossicida* from an exclusively agricultural problem into a global emerging infectious disease threat, necessitating urgent attention from infectious disease specialists [7]. The same virulence determinants that drive systemic hemorrhage and necrosis in fish may facilitate invasion of the human blood−brain barrier, yet no dedicated therapeutic pipeline currently addresses this dual-host pathogen [6,7].

The challenge is compounded by the global crisis of antimicrobial resistance (AMR). Traditional antibiotics exert selective pressure that drives the evolution and spread of resistant strains, a concern particularly acute for *Pseudomonas* species, which are notoriously adept at acquiring resistance determinants [8]. In this context, conventional bactericidal or bacteriostatic approaches risk accelerating resistance, especially as *P. plecoglossicida* transitions between aquatic reservoirs and human hosts. Consequently, alternative strategies that disarm pathogens without directly inhibiting their growth have gained considerable attention [8,9]. Anti−virulence therapy—which targets quorum sensing, biofilm formation, and toxin production—offers a promising paradigm. By disrupting bacterial communication and virulence factor expression rather than bacterial viability, such approaches minimize the selective pressure that drives AMR, potentially preserving long−term efficacy.

The search for alternative therapies—particularly from traditional Chinese medicine (TCM)—must be reframed not merely as an agricultural necessity but as an emerging public health imperative [10]. Among these, *Peganum harmala* (Syrian Rue), a medicinal plant traditionally used for its antimicrobial and anti-inflammatory properties, is notable for its high concentration of β−carboline alkaloids, particularly harmine and harmaline, which exhibit diverse pharmacological activities including direct antibacterial and immunomodulatory effects [11,12]. Harmine, a principal active constituent, shows promise against challenging pathogens [13]. It can disarm bacteria by inhibiting quorum sensing in *Pseudomonas aeruginosa* [14], and attenuate virulence by suppressing the type III secretion system (T3SS) in *Salmonella* Typhimurium, improving survival in infected mice [15]. Furthermore, harmine derivatives have been shown to enhance macrophage−mediated bacterial clearance, acting as immunomodulatory adjuvants in models of drug−resistant *Acinetobacter baumannii* pneumonia [16]. Despite these broad activities, the potential of harmine against major aquatic pathogens remains largely unexplored.

To address the dual threat posed by *P. plecoglossicida*—as an aquaculture pathogen with devastating economic impact and as an emerging zoonotic agent capable of causing life-threatening meningitis—we screened 30 antimicrobial TCM monomers and identified harmine as a potent agent against *P. plecoglossicida*. In this study, we aimed to (1) evaluate the direct antibacterial activity of harmine against *P. plecoglossicida* in vitro; (2) assess the therapeutic efficacy of harmine in an ayu infection model in vivo; and (3) elucidate the molecular mechanism by which harmine enhances host immunity, with a focus on identifying its direct protein target and characterizing the downstream signaling pathways involved.

## 2. Materials and Methods

### 2.1. Animal and Pathogen

Juvenile ayu fish (*Plecoglossus altivelis*), measuring 5−10 cm in length, were sourced from the Ninghai Aquaculture Farm (Ningbo, China). Prior to experimentation, fish were acclimatized for 14 days in a recirculating aquaculture system with UV−sterilized water (254 nm) and mechanical and biological filtration to minimize exposure to environmental pathogens. Water quality parameters were maintained within standard ranges (temperature 20 ± 1 °C, pH 7.8 ± 0.2, ammonia < 0.02 mg/L). The pathogenic bacterium *P*. *plecoglossicida* was used for infection. All animal experiments were conducted in compliance with China’s Laboratory Animal Management Law and were approved by the Animal Ethics Committee of Ningbo University (protocol code: Approval No. AEWC−11140 and date of approval: 22 February 2024).

### 2.2. Compound Screening

A chemical library of 30 previously reported antibacterial compounds derived from traditional Chinese medicine (TCM) was screened for activity against *P*. *plecoglossicida*. All compounds were tested at a final concentration of 25 µM, with each assay performed in triplicate. Screening was performed in 96-well plates containing a co−incubation of the diluted TCM monomer and bacterial suspension. OD_595_ measurements were taken every 2 h using a SpectraMax Paradigm microplate reader (Molecular Devices, San Jose, CA, USA) to monitor bacterial growth. Antibacterial activity was assessed by generating and analyzing time-dependent growth curves.

### 2.3. Evaluating Therapeutic Effects of Harmine on Survival and Tissue Bacterial Load in P. plecoglossicida-Infected Ayu

To evaluate the effect of harmine on survival in *P. plecoglossicida*-infected ayu, bacteria were revived from −80 °C, cultured overnight in TSB at 28 °C, and subcultured to logarithmic phase. After microscopic enumeration, 175 ayu were divided into five groups (*n* = 25). Fish were intraperitoneally injected with *P. plecoglossicida* (5000 CFU/g). In treatment groups, harmine (50 or 100 μg/g) was administered 4 h post-infection; in prevention groups, harmine was administered prior to infection. Controls received PBS. Survival was recorded every 12 h.

A standard colony-forming unit (CFU) assay was used to quantify the bacterial load in the liver, spleen, head kidney, and blood of *P. plecoglossicida*−infected ayu at 24 h post−final drug administration [17]. At designated time points post−infection, liver tissue was aseptically excised and weighed. The tissue was then homogenized in a known volume of sterile 1 × phosphate−buffered saline (PBS) using a tissue grinder. Serial 10-fold dilutions of the homogenate were prepared, and 100 µL aliquots from selected dilutions were spread onto Tryptic Soy Agar (TSA) plates. After incubation at 28 °C for 24−48 h, the number of viable bacterial colonies was counted. The bacterial load was calculated and expressed as Log_10_ CFU per gram of liver tissue (Log_10_ CFU/g).

### 2.4. Evaluating In Vitro Antibacterial Activity of Harmine Against P. plecoglossicida

Following a standard microbroth dilution protocol [18], the minimum inhibitory concentration (MIC) of harmine against *P. plecoglossicida* was determined. The strain was first revived from a glycerol stock stored at −80 °C and grown overnight in Tryptic Soy Broth (TSB) at 28 °C with shaking. Subsequently, this culture was adjusted to ~1 × 10^8^ CFU/mL in fresh TSB before seeding 96−well plates with 100 µL aliquots. Harmine, dissolved in DMSO (final DMSO concentration < 1%), was added to achieve a twofold serial dilution with final concentrations ranging from 20 to 200 µg/mL. The assay included ciprofloxacin (0.5 µg/mL) as a positive control and medium-only wells as a negative control, with all conditions run in triplicate. Following a 24 h incubation at 28 °C with 150 rpm shaking, growth was assessed by measuring OD_595_.

### 2.5. Biofilm Formation Assay

Biofilm formation by *P. plecoglossicida* in the presence of harmine (50 and 100 µg/mL) was evaluated via crystal violet staining [17]. Bacterial suspension (1 × 10^6^ CFU/mL, 100 µL/well) was incubated statically at 28 °C for 24 h in a 96−well plate. After washing to remove planktonic cells, adherent biofilms were stained with 0.4% crystal violet, washed, and the bound dye was eluted with 33% glacial acetic acid. Biofilm biomass was quantified by measuring absorbance at 570 nm.

### 2.6. Bacterial Viability and Membrane Integrity Assay

Bacterial viability following harmine treatment was assessed by dual-fluorescence staining. *P. plecoglossicida* (1 × 10^6^ CFU/mL) was treated with harmine (50 or 100 µg/mL) or ciprofloxacin (0.5 µg/mL, positive control) for 3 h at 28 °C [17]. After washing, cells were stained with Hoechst 33342 (permeant, stains all DNA) and propidium iodide (PI; impermeant, stains dead/damaged cells) for 20 min in the dark. Fluorescence microscopy was used to distinguish viable cells (blue Hoechst only) from non-viable cells (red PI fluorescence), thereby evaluating membrane damage and cell death.

### 2.7. Cell Thermal Shift Assay

A cellular thermal shift assay (CETSA) was employed to assess harmine binding to ayu PRDX6 [19]. Liver protein extracts (100 μg) were incubated with 3 μM harmine or vehicle and subjected to a temperature gradient (45–70 °C, 5 min). Proteins were separated by SDS−PAGE, transferred to PVDF membranes, blocked, and probed overnight at 4 °C with anti−PRDX6 (in-house) and anti-β-tubulin (Beyotime) antibodies. Following incubation with an HRP-conjugated secondary antibody, bands were detected by ECL. Thermal stability curves were generated from band intensities, and a shift toward higher stability in harmine−treated samples indicated direct binding.

### 2.8. Isolation of Ayu MO/Mφ

MO/Mφ were isolated using a Ficoll-Paque density gradient [1]. Tissue was passed through a 100-μm strainer to obtain a single-cell suspension, which was overlaid onto Ficoll-Paque PLUS (1.077 g/mL) and centrifuged (500× *g*, 25 min, RT, low brake). The mononuclear leukocyte interphase was harvested, washed twice in DMEM (500× *g* for 8 min, then 250× *g* for 5 min), and resuspended in complete DMEM. Viable cells were counted by trypan blue exclusion and adjusted to 2 × 10^7^ cells/mL. After 6–8 h of adherent culture, those that adhered to the bottom of the culture dish were the MO/Mφ.

### 2.9. CCK8 Assay

Cytotoxicity was determined with a Cell Counting Kit-8 (CCK-8) assay. Ayu MO/MΦ and other detected cells (1 × 10^4^ cells/well) were adhered overnight in a 96-well plate, then treated with serial dilutions of harmine (0–200 µg/mL) for 48 h. CCK-8 reagent (10 µL/well) was added, and plates were incubated for 4 h at 28 °C. Absorbance at 450 nm was recorded on a SpectraMax Paradigm reader. Cell viability was calculated as: Viability (%) = [(sample − blank)/(control − blank)] × 100, where “control” refers to untreated cells and “blank” to medium only.

### 2.10. RNA Isolation and Quantitative Real-Time PCR Analysis

The detected gene’s mRNA levels were measured by RT-qPCR. Total RNA was extracted from ayu liver, and cDNA was synthesized from 1 µg RNA. RT-qPCR reactions (20 µL) contained SYBR Premix Ex Taq, gene-specific primers (0.8 µL each, 10 µM), 2 µL cDNA, and water. Amplification conditions were: 95 °C for 5 min; 40 cycles of 95 °C for 30 s, 60 °C for 30 s, 72 °C for 30 s; followed by melt curve analysis. Target gene expression (Table 1) was normalized to β-actin using the 2^−ΔΔCt^ method [1,20]. All samples were run in technical triplicates across three biological replicates.

### 2.11. Macrophage/Monocyte Bactericidal Activity Assay

To quantify macrophage bactericidal activity, a dual-calibration strategy combining colony-forming unit (CFU) enumeration with *gyrB*-targeted quantitative PCR (qPCR) was established for *Pseudomonas plecoglossicida* according to a previous report [21]. A standard curve linking *gyrB* copy number to CFU was generated using serial dilutions of mid-log-phase bacteria, with absolute quantification enabled by a plasmid standard containing the *gyrB* amplicon (efficiency 90–110%, R^2^ ≥ 0.99). Macrophages infected at the indicated multiplicity of infection were treated with gentamicin (100 μg/mL, 1 h) to eliminate extracellular bacteria, then lysed at specified time points for DNA extraction. qPCR with *gyrB*-specific primers (validated against host genomic DNA) was performed, and sample Ct values were converted to *gyrB* copy numbers using the plasmid standard, then to equivalent CFU via the pre-established correlation. Bacterial survival was calculated as (equivalent CFU at time 2 h/equivalent CFU at 0 h) × 100%, with bactericidal activity expressed as 100% minus survival.

### 2.12. Phagocytosis Assay

Phagocytosis by ayu monocytes/macrophages (MO/MΦ) in the presence of harmine was assessed via flow cytometry. Adherent cells (2 × 10^7^ cells/mL) were infected with BacLight™ Red-labeled *P. plecoglossicida* (MOI = 10, 1 h) [21]. Following removal of non-internalized bacteria, cells were treated with harmine (50 or 100 µg/mL) for 12 h and then with gentamicin (100 µg/mL, 1 h) to kill extracellular bacteria. After washing and resuspension, ≥10,000 cells per sample were analyzed on a BD FACSCanto II. Data were evaluated with FlowJo VX10, and phagocytic activity was reported as the percentage of positive cells and/or the mean fluorescence intensity (MFI).

### 2.13. Apoptosis Assay

To quantify apoptosis in *P. plecoglossicida*-infected ayu MO/MΦ, an Annexin V-FITC and PI dual-staining assay was performed following a modified published method [21]. Cells (2 × 10^6^) were washed, resuspended in binding buffer, and stained with Annexin V-FITC for 15 min in the dark, followed by PI for 5 min. Flow cytometry was conducted immediately, and the resulting data were analyzed with FlowJo VX10 software (VX10.6.2, TreeStar, Ashland, OR, USA) to categorize cells as viable (Annexin V^−^/PI^−^), early apoptotic (Annexin V^+^/PI^−^), or late apoptotic/necrotic (Annexin V^+^/PI^+^).

### 2.14. Western Blot

Protein expression was analyzed by Western blotting. Briefly, lysates from cells or tissues were prepared in RIPA buffer with protease/phosphatase inhibitors, clarified by centrifugation, and quantified (BCA assay). Proteins (20–40 µg) were separated by SDS-PAGE (8–12% gels), transferred to PVDF membranes, and blocked in 5% milk or BSA. Membranes were probed overnight at 4 °C with primary antibodies (including β-actin for normalization), followed by HRP-conjugated secondary antibodies. Blots were developed with ECL, imaged (ChemiDoc, Bio-Rad), and quantified (ImageJ2). Target protein expression was normalized to loading controls.

### 2.15. Enzyme Activity Assay

To evaluate antioxidant capacity and immune-related enzyme activities in ayu (*P. altivelis*) serum, spectrophotometric assays were performed using commercial kits. Blood samples were collected from the caudal vein using non-heparinized syringes and allowed to clot at 4 °C for 2 h. Serum was separated by centrifugation at 3000× *g* for 10 min at 4 °C and stored at −80 °C until analysis. All assays were performed in triplicate with appropriate blank controls, and results were normalized to serum volume.

For liver damage markers (AST, ALT, ALP): AST and ALT activities were measured using commercial assay kits (Beyotime, China; Cat. No. P2715S for AST, P2711S for ALT). Briefly, 20 µL of serum was mixed with 100 µL of substrate solution containing L-aspartate and α-ketoglutarate (AST) or L-alanine and α-ketoglutarate (ALT). After incubation at 37 °C for 30 min, 100 µL of 2,4-dinitrophenylhydrazine (DNPH) solution was added to terminate the reaction and form hydrazone derivatives. Following a further 20 min incubation at 37 °C, 1.0 mL of 0.4 mol/L NaOH was added, and absorbance was measured at 510 nm using a SpectraMax Paradigm microplate reader. Enzyme activity was calculated based on a standard curve prepared with pyruvate standards (0–200 µmol/L). One unit of AST or ALT activity was defined as the amount of enzyme that catalyzes the formation of 1 µmol of pyruvate per minute at 37 °C. Activities were expressed as units per liter (U/L) of serum.

For alkaline phosphatase (ALP): ALP activity was determined using a commercial kit (Beyotime, Cat. No. P0321S). In brief, 10 µL of serum was mixed with 100 µL of reaction buffer containing 5 mmol/L p-nitrophenyl phosphate (pNPP) in 1 mol/L diethanolamine buffer (pH 9.8). After incubation at 37 °C for 15 min, the reaction was terminated by adding 100 µL of 0.5 mol/L NaOH. Absorbance was measured at 405 nm. A standard curve was generated using p-nitrophenol standards (0–100 µmol/L). One unit of ALP activity was defined as the amount of enzyme that hydrolyzes 1 µmol of pNPP to p-nitrophenol per minute at 37 °C. ALP activity was expressed as U/L serum.

For antioxidant enzyme activities (SOD, CAT): Total SOD activity was measured using a water-soluble tetrazolium salt (WST-1) assay kit (Beyotime, Cat. No. S0101S). The assay is based on the reduction of WST-1 to a water-soluble formazan dye by superoxide anions generated by xanthine oxidase. Serum samples (20 µL) were mixed with 200 µL of reaction solution containing WST-1 (0.5 mmol/L) and xanthine oxidase (0.1 U/mL). The reaction was carried out at 37 °C for 20 min, and absorbance was read at 450 nm. Inhibition rate was calculated as:

$$\mathrm{Inhibition}\;(\%)=\frac{\left(A_{\mathrm{blank}1}-A_{\mathrm{blank}2})-(A_{\mathrm{sample}}-A_{\mathrm{control}}\right)}{\left(A_{\mathrm{blank}1}-A_{\mathrm{blank}2}\right)}\times 100$$

where $A_{\mathrm{blank}1}$: reaction without sample, $A_{\mathrm{blank}2}$: reaction without xanthine oxidase, $A_{\mathrm{sample}}$: sample reaction, and $A_{\mathrm{control}}$: sample reaction without xanthine oxidase.

One unit of SOD activity was defined as the amount of enzyme required to achieve 50% inhibition of the WST-1 reduction reaction. SOD activity was expressed as U/mL serum.

CAT activity was assayed using a colorimetric kit based on ammonium molybdate (Beyotime, Cat. No. S0051). The principle involves the decomposition of hydrogen peroxide (H_2_O_2_) by catalase, with residual H_2_O_2_ forming a yellow complex with ammonium molybdate. Briefly, 20 µL of serum was mixed with 100 µL of H_2_O_2_ solution (65 µmol/L) and incubated at 25 °C for 60 s. The reaction was terminated by adding 100 µL of ammonium molybdate (32.4 mmol/L). Absorbance was measured at 405 nm. CAT activity was calculated as:

$$\begin{array}{l} \mathrm{CAT}\;\mathrm{Activity}\;(U/\mathrm{mL})\\ =\frac{\left(A_{control}-A_{sample}\right)\times Total\;volume\left(mL\right)\times Dilution\;factor}{Reaction\;time\left(s\right)\times Sample\;volume\left(mL\right)\times Molar\;extinction\;coefficient\left(0.0436\;{µmol}^{-1}{cm}^{-1}\right)} \end{array}$$

One unit of CAT activity was defined as the amount of enzyme that decomposes 1 µmol of H_2_O_2_ per second at 25 °C. Results were expressed as U/mL serum.

### 2.16. Lipid Peroxidation Marker

Malondialdehyde (MDA), a marker of lipid peroxidation, was measured using a thiobarbituric acid (TBA) reaction kit (Beyotime, Cat. No. S0131S) [20]. In this assay, MDA reacts with TBA under acidic conditions to form a pink-colored complex. Serum (100 µL) was mixed with 200 µL of TBA working solution (containing 0.375% TBA in 0.25 mol/L HCl) and heated at 100 °C for 15 min. After cooling to room temperature, the mixture was centrifuged at 1000× *g* for 10 min to remove precipitates. The absorbance of the supernatant was measured at 532 nm. A standard curve was prepared using MDA standards (0–10 µmol/L). MDA concentration was calculated as:

$$\mathrm{MDA}\;(µ\mathrm{mol}/L)=\frac{A_{\mathrm{sample}}-A_{\mathrm{blank}}}{A_{\mathrm{standard}}-A_{\mathrm{blank}}}\times C_{\mathrm{standard}}$$

where $C_{\mathrm{standard}}$ is the concentration of the MDA standard (10 µmol/L). Results were expressed as µmol/L serum.

### 2.17. DARTS Assay

The Drug Affinity Responsive Target Stability (DARTS) assay was employed to identify protein targets of harmine, based on the principle that small-molecule binding stabilizes target proteins against proteolytic degradation. Macrophages/monocytes were isolated and cultured as previously described [20]. Eight hours post-infection with *P. plecoglossicida*, the cells were washed with ice-cold PBS, harvested by scraping, and lysed in ice-cold lysis buffer supplemented with protease inhibitors. After centrifugation (16,000× *g*, 15 min, 4 °C), the protein concentration of the supernatant was quantified by BCA assay. Aliquots containing equal protein amounts (10 µg) were incubated with 3.0 µM harmine or DMSO vehicle for 30 min at 4 °C. Samples were then subjected to limited proteolysis across a temperature gradient (45–70 °C in 5 °C increments, 5 min per step). Following a second centrifugation, the supernatant was mixed with 5× SDS-PAGE loading buffer, denatured (95 °C, 5 min), and resolved by SDS-PAGE. Proteins were visualized using a Fast Silver Stain Kit (Beyotime). Differential bands between the harmine- and DMSO-treated groups were excised, trypsin-digested, and identified by liquid chromatography-tandem mass spectrometry (LC-MS/MS).

### 2.18. Co-IP Assay

Co-immunoprecipitation (co-IP) was performed according to an established protocol [22,23]. Cells were lysed in NP-40 buffer (Beyotime) with protease inhibitors (Roche), and cleared lysates were pre-cleared with Protein A/G Magnetic Beads (Thermo Fisher Scientific, Cleveland, OH, USA). Pre-cleared samples (500–1000 µg) were incubated overnight at 4 °C with a specific primary antibody or isotype control. Immune complexes were captured with fresh beads, washed, and eluted by boiling in 2× SDS-PAGE buffer. Input and immunoprecipitated proteins were separated by SDS-PAGE and analyzed by Western blot. Blots were developed with ECL and imaged on a ChemiDoc system (Bio-Rad).

### 2.19. siRNA Transfection into Ayu MO/Mφs

Ayu MO/Mφs were seeded in 12-well plates at a density of 1 × 10^6^ cells per well in DMEM medium supplemented with 10% fetal bovine serum (FBS) and incubated at 28 °C for 24 h. On the day of transfection, the culture medium was replaced with antibiotic-free DMEM without FBS. For each well, 1 µL of 20 µM siRNA (*PRDX6* or negative control) was diluted in 50 µL Opti-MEM, and 1.5 µL of Lipofectamine RNAiMAX was diluted separately in 50 µL Opti-MEM; the two solutions were mixed gently and incubated at room temperature for 15 min to form siRNA–lipid complexes. The 100 µL complex mixture was added dropwise to each well, and cells were incubated at 28 °C for 6 h. Subsequently, the transfection medium was replaced with fresh complete DMEM containing 10% FBS and antibiotics, and cells were cultured for an additional 48 h before harvesting for downstream analyses (qRT-PCR and Western blot). A scrambled siRNA was used as a negative control, and transfection efficiency was assessed using FAM-labeled control siRNA.

### 2.20. ROS Level Assay

To assess cellular ROS levels, two distinct fluorescent probes were used. For total intracellular ROS measurement, cells were washed with PBS after treatment and incubated with 10 μM DCFH-DA (Beyotime) for 20 min at 37 °C under 5% CO_2_. Cells were then washed three times with PBS to remove excess probe, trypsinized to obtain a single-cell suspension, and immediately analyzed by flow cytometry [24]. For specific detection of mitochondrial ROS (mROS), cells were stained with 5 μM MitoSOX Red (Beyotime) for 10 min at 37 °C in the dark after treatment and PBS washing. Cells were then washed twice with PBS, trypsinized, and analyzed by flow cytometry to assess mitochondrial superoxide production [21].

### 2.21. Statistical Analysis

Analyses were performed in GraphPad Prism 9.0 (GraphPad, Boston, MA, USA). For comparisons between two groups, Student’s *t*-test was applied; for comparisons among three or more groups, one-way ANOVA was used. Survival was analyzed by the Kaplan–Meier method with log-rank testing. Statistical significance (two-tailed) was set at *p* < 0.05, unless otherwise indicated. Results are expressed as mean ± standard error of the mean (SEM) from three independent experiments, with figure error bars corresponding to SEM. Asterisks indicate significance versus the bacterial control group: * *p* < 0.05, ** *p* < 0.01, *** *p* < 0.001.

## 3. Results

### 3.1. Harmine Exhibits a Significant Inhibitory Effect on P. plecoglossicida

Owing to the lack of effective therapeutics for *P. plecoglossicida*, a significant pathogen in aquaculture [4,5,25], we screened 30 traditional Chinese medicine (TCM) monomers with known antibacterial activities (Figure S1A). Growth curve assays identified harmine as the only compound with significant inhibitory activity against *P. plecoglossicida* (Figure S1B).

### 3.2. Harmine Exerts Its Direct Antibacterial Activity by Inhibiting Bacterial Biofilm Formation and Raising Bacterial Cell Membrane Permeability

**Figure 1:**
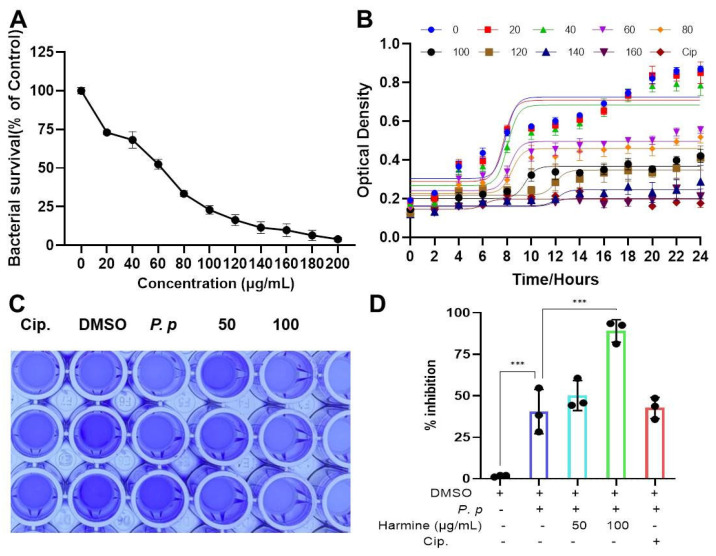
Harmine inhibits the proliferation and biofilm formation of *P. plecoglossicida*. (**A**) Harmine kills the *P. plecoglossicida* in a dose-dependent manner. (**B**) The effect of different concentrations of harmine on the growth curve of *P. plecoglossicida* (mean ± SEM). (**C**,**D**) The effect of harmine on the biofilm formation of *P. plecoglossicida*. *** *p* < 0.0001 compared to *P. plecoglossicida* group. To elucidate the potential antibacterial mechanisms of harmine in vitro, we assessed its effects on two critical virulence phenotypes: biofilm formation and membrane integrity [17]. Our findings demonstrate that harmine significantly inhibits biofilm formation (**C**,**D**) and dose-dependently increases bacterial membrane permeability (Figure 2). This dual action—compromising both structural community defenses and individual cell integrity—likely underpins its efficacy in promoting bacterial clearance.

To further characterize the anti-*P. plecoglossicida* activity of harmine, we determined its minimum inhibitory concentration (MIC) and its effect on bacterial growth kinetics. The growth curves, monitored by measuring OD_595_ at 2 h intervals over 24 h, revealed that harmine dose-dependently inhibited the proliferation of the MIC_50_ of *P. plecoglossicida* with a half minimum inhibitory concentration (MIC_50_) of about 62.3 μg/mL (Figure 1A). Furthermore, the maximum bacterial density achieved during the logarithmic phase was significantly reduced in a similar trend within the tested concentration range (20–200 μg/mL) (Figure 1B).

**Figure 2:**
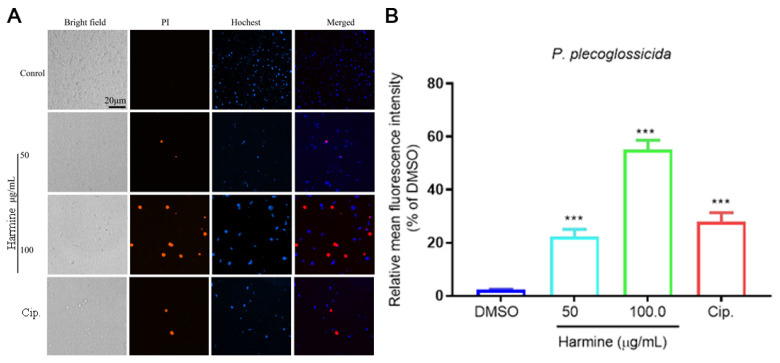
Harmine enhances the membrane permeability of *P. plecoglossicida* in a dose-dependent manner. (**A**,**B**) The effect of harmine on bacterial membrane permeability (**A**) and their statistics (**B**). *** *p* < 0.0001 compared to DMSO group.

### 3.3. Harmine Significantly Improves the Survival of Ayu and Reduces Bacterial Load in P. plecoglossicida-Infected Ayu

**Figure 3:**
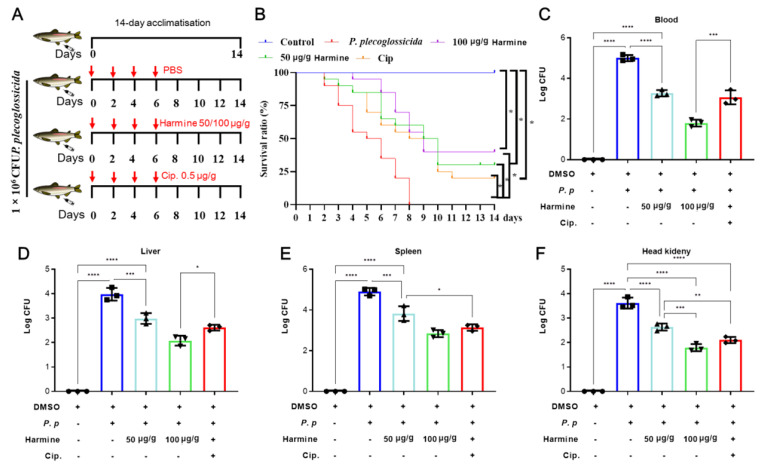
The impact of different concentrations of harmine treatment on the ayu survival rate and bacterial load following *P. plecoglossicida* infection. (**A**) Experimental grouping and processing protocols used in this study. (**B**) Fish survival was analyzed using Kaplan–Meier survival analysis combined with the log-rank test. (**C**–**F**) The effects of harmine on the bacterial load of ayu blood (**C**), liver (**D**), spleen (**E**), and head kidney (**F**). * *p* < 0.05, ** *p* < 0.01, *** *p* < 0.0001 and **** *p* < 0.0001 compared to *P. plecoglossicida* group.

To further evaluate the therapeutic potential of harmine against *P. plecoglossicida* infection in vivo, the effect of harmine on the ayu survival rate at day 14 and on tissue bacterial load at day 7 (Figure 3A) was examined. Following drug administration, ayu exhibited no abnormal swimming behavior, tremors, or loss of balance. The results showed that the infected ayu exhibited lethargy, loss of appetite, abdominal distension, and hemorrhagic lesions on the body surface, consistent with typical BHA, and all fish in the infected model group succumbed by day 8, with gross examination revealing abdominal swelling with hemorrhagic ascites, multifocal hemorrhages in the liver and kidney, and splenomegaly. Therapeutic administration of harmine at both tested concentrations significantly improved the survival rate (30% and 40%, respectively) until day 14, an effect that surpassed the protection offered by the ciprofloxacin (Cip) group (Figure 3B). Meanwhile, harmine significantly reduced the bacterial load in the blood, liver, spleen and head kidney (Figure 3C–F).

### 3.4. Changes in Serum Biochemical Indices for P. plecoglossicida-Infected Ayu After Harmine Treatment

**Figure 4:**
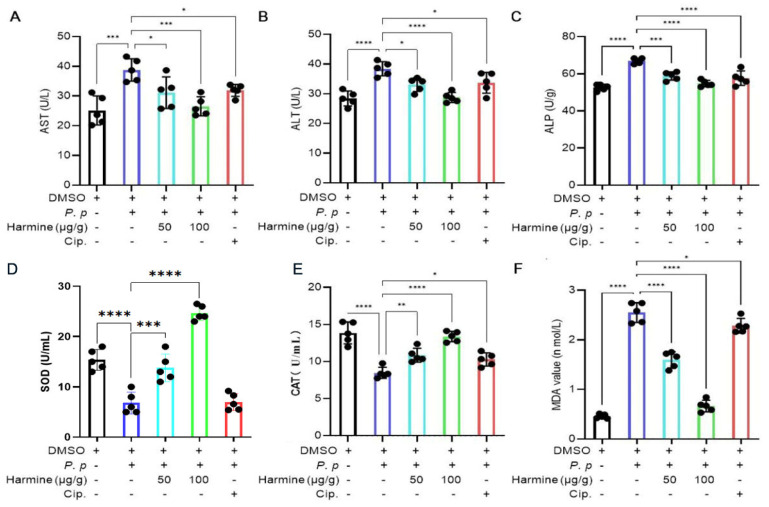
The impact of harmine on serum liver damage markers and antioxidant responses following *P. plecoglossicida* infection. (**A**) AST, (**B**) ALT, (**C**) ALP, (**D**) SOD, (**E**) CAT, (**F**) MDA. * *p* < 0.05, ** *p* < 0.01, *** *p* < 0.0001 and **** *p* < 0.0001 compared to *P. plecoglossicida* group.

Bacterial infection leads to dramatic alternations in serum biochemical indices, including immune and antioxidant enzyme activities [26,27]. Therefore, we further investigated the effects of harmine on these enzymes involved in bacteriostatic activity. The results showed that harmine significantly reduced *P. plecoglossicida*-infected elevation of AST, ALT, and alkaline phosphatase (ALP) (Figure 4A–C), three biomarkers for liver damage. Moreover, harmine also enhanced the antioxidant capacity of serum, as indicated by raising SOD and CAT activity, and reduced MDA levels compared to the *P. plecoglossicida*-infected group (Figure 4D,E).

### 3.5. Harmine Reduces P. plecoglossicida-Tissue Inflammation in Ayu

**Figure 5:**
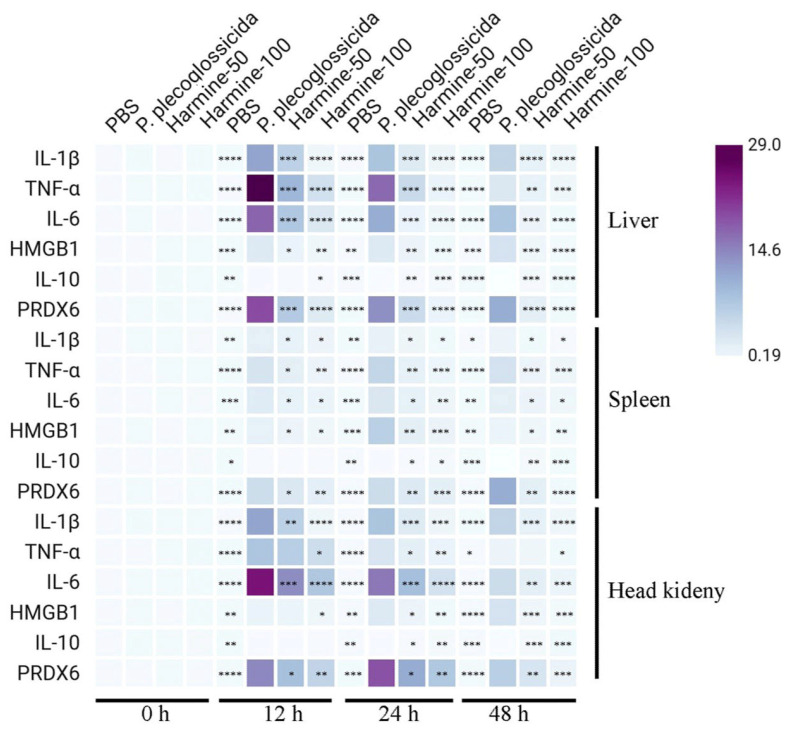
Harmine modulates tissue-specific gene mRNA expression in *P. plecoglossicida*-infected ayu. mRNA levels in the liver, spleen, and head kidney were measured by RT-qPCR. Results are shown as mean ± SEM of three biological replicates and are representative of a minimum of six independent experiments. Statistical comparisons were performed using two-way ANOVA followed by post hoc tests. Asterisks denote significant differences from the infection-only control: * *p* < 0.05, ** *p* < 0.01, *** *p* < 0.001, **** *p* < 0.0001.

In mammals, harmine effectively suppresses inflammatory responses [28]. Studies have shown that harmine inhibits inflammation by abrogating NF-κB signaling [29,30,31]. To determine whether harmine exerts similar immunomodulatory effects in fish, we measured the expression levels of inflammatory factors—TNF-α, IL-1β, IL-6, PRDX6, IL-10, and HMGB1—in ayu liver, spleen, and head kidney at 12, 24, and 48 h post-treatment. Harmine induced a dose-dependent downregulation of pro-inflammatory factors (*TNF-α*, *IL-1β*, *IL-6*, *PRDX6*, and *HMGB1*) across all tissues. Conversely, it significantly upregulated the anti-inflammatory cytokine *IL-10* in a dose-dependent manner, with the most pronounced effect observed at 24 h (Figure 5).

### 3.6. PRDX6 Is the Potential Target of Harmine

**Figure 6:**
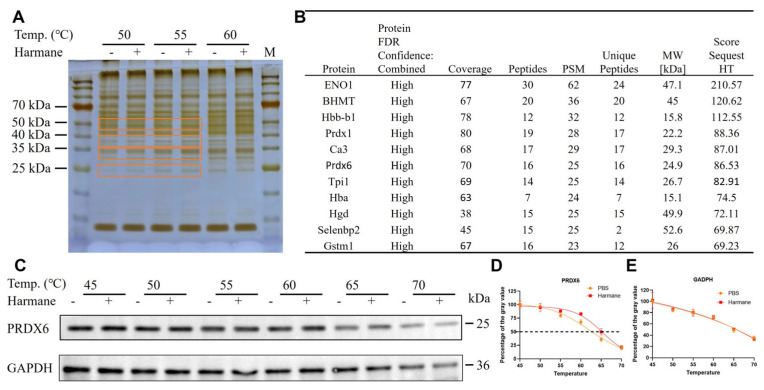
PRDX6 is identified as the potential target of harmine based on DARTS and cell thermal shift assay. (**A**) Identification of the potential target of harmine using DARTS analysis. (**B**) The top 11 identified protiens and their corresponding information. (**C**) Thermal shift profile of PRDX6 upon harmine by CETSA. (**D**,**E**) Quantitative analysis of PRDX6 and GAPDH expression levels by ImageJ2 software.

Given the promising antibacterial activity of harmine, we employed a Drug Affinity Responsive Target Stability (DARTS) assay to identify its direct molecular targets. Following limited thermal proteolysis and SDS-PAGE, silver staining revealed a distinct protein band exhibiting enhanced stability in the harmine-treated group, with three apparent molecular weights near 25, 35, and 55 kDa (Figure 6A). This differentially stabilized band was excised and analyzed by mass spectrometry, which identified 11 candidate target proteins (Figure 6B). Based on its molecular weight alignment with the observed band and its established role in oxidative stress response, peroxiredoxin 6 (PRDX6) was prioritized for validation. A subsequent cellular thermal shift assay (CETSA) confirmed a direct, dose-dependent interaction between harmine and PRDX6 in intact cells (Figure 6C–E), demonstrating that harmine binding increases the thermal stability of PRDX6.

### 3.7. Harmine Exhibits Its In Cellulo Antibacterial Activity via Inducing mROS Production by Binding to PRDX6

To further investigate the harmine-mediated antibacterial mechanisms in cellulo, we first evaluated the cytotoxicity of harmine to six normal cell lines after incubation with various concentration gradients for 72 h to determine the safe concentration of harmine for further analysis. The results showed that harmine exerts no obvious toxicity to any detected cell lines when its concentration is below 100 μg/mL (Figure S2); thus, we chose the concentrations of 50 and 100 μg/mL for our following in cellulo assays.

**Figure 7:**
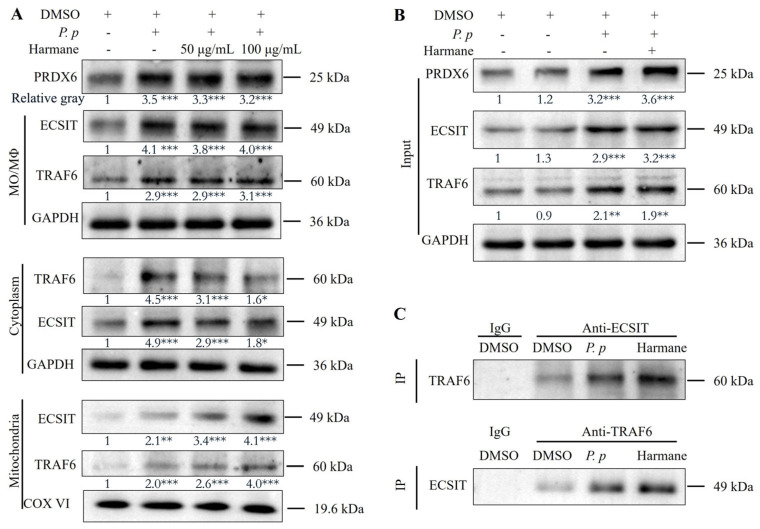
Harmine promotes the translocation of TRAF6 and ECSIT from the cytoplasm to the mitochondria via blocking TRAF6 −PRDX6 interaction. (**A**) The effects of harmine on the alternations alterations of PRDX6, ECSIT and TRAF6 in MO/MΦ. (**B**) The effect of harmine on the translocation of TRAF6 and ECSIT from the cytoplasm to the mitochondria. (**C**) The effect of harmine on TRAF6−ECSIT interaction in MO/MΦ. Data represent mean ± SEM (*n* = 3); significance vs. bacterial-only control: * *p* < 0.05, ** *p* < 0.01, and *** *p* < 0.001.

A previous study revealed that PRDX6 negatively affects bactericidal activity against *Salmonella typhimurium* and NF-κB activity by interrupting the TRAF6-ECSIT complex, reducing the translocation of the PRDX6-TRAF6 complex from the cytoplasm to the mitochondria, and thus reducing TRAF6-ECSIT complex-mediated mROS production in human monocytic leukemia THP-1 cells [32]. To explore whether harmine exhibits its anti-*P. plecoglossicida* activity via targeting this signaling pathway, we detected the effects of harmine on expression, translocation, and interactions among TRAF6/ECSIT/PRDX6 by SDS-PAGE and co-IP assay. The results showed that harmine could not reduce the elevations of three proteins induced by *P. plecoglossicida* infection (Figure 7A), but it promoted the translocation of TRAF6 and ECSIT from the cytoplasm to the mitochondria (Figure 7B) and thus enhanced the interaction between TRAF6 and ECSIT via binding PRDX6 (Figure 6C–E and Figure 7C).

**Figure 8:**
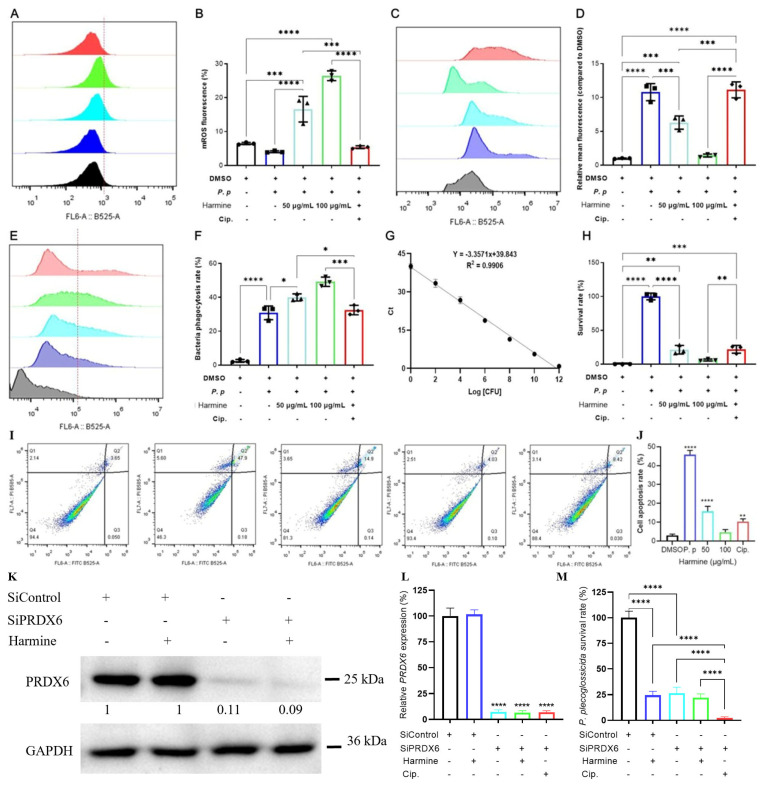
Harmine effects on ROS/mROS levels, phagocytic and bacterial capacity and apoptosis of ayu MO/MΦ via targeting PRDX6. (**A**–**D**) ROS and mROS levels. (**E**) Phagocytosis assay. (**F**) Bacterial uptake quantification. (**G**) *P. plecoglossicida* standard curve (*gyrB* RT−qPCR). (**H**) Bactericidal activity. (**I**,**J**) Apoptosis analysis by flow cytometry and statistics. (**K**,**L**) Evaluation of the knockdown effect of SiPRDX6: Western blot (**K**) and RT−qPCR (**L**). (**M**) The effect of PRDX6 level on the efficacy of harmine-mediated bactericidal activity in ayu MO/MΦ. Data represent mean ± SEM (*n* = 3); significance vs. bacterial-only control: * *p* < 0.05, ** *p* < 0.01, *** *p* < 0.001 and **** *p* < 0.0001.

The effects of harmine on mROS and total ROS production were assessed using flow cytometry. Harmine treatment significantly increased mitochondrial ROS (mROS) while concurrently reducing total cellular ROS levels (Figure 8A–D). Although this pattern may appear counterintuitive, it is consistent with the compartmentalized nature of ROS regulation: mROS is generated primarily via the mitochondrial electron transport chain and serves as a localized signaling burst essential for antimicrobial function, whereas total ROS reflects contributions from multiple sources, including NADPH oxidases and other non-mitochondrial compartments [33,34]. The observed increase in mROS aligns with the intracellular bacterium−induced ROS response, further supporting the characterization of *P. plecoglossicida* as a facultative intracellular pathogen [35]. Moreover, the simultaneous reduction in total ROS can be attributed to harmine-induced activation of compensatory antioxidant mechanisms (upregulation of catalase, Figure 4D,E) and potential suppression of non-mitochondrial ROS sources, consistent with the immunomodulatory rather than pro-oxidant profile of the compound. Collectively, these data support the identification of PRDX6 as a key molecular target through which harmine exerts its protective effects against *P. plecoglossicida* infection via raising mROS levels and maintaining redox homeostasis in MO/MΦ.

### 3.8. Harmine Increases Phagocytosis and Bactericidal Activity and Reduces P. plecoglossicida-Induced Apoptosis of Ayu MO/MΦ

To determine whether harmine modulates the innate immune function of ayu macrophages (MO/MΦ), we assessed its effect on bacterial phagocytosis. Harmine significantly enhanced phagocytic activity at concentrations of 50 and 100 μg/mL in therapeutic treatment regimens compared to controls (Figure 8E,F) in a dose−dependent mode.

We next evaluated the effect of harmine on the bactericidal capacity of MO/MΦ against *P. plecoglossicida*. Compared to the infection-only group, bacterial survival was significantly reduced in harmine−treated groups (50 μg/mL: 21.28 ± 3.61%; 100 μg/mL: 6.17 ± 1.15%), with efficacy approaching that of the ciprofloxacin control (22.07 ± 3.08%) (Figure 8G,H). Notably, therapeutic administration of harmine potentiated macrophage-mediated bacterial killing more effectively than prophylactic administration. Our previous study demonstrated that *P. plecoglossicida* infection induces MO/MΦ apoptosis, exacerbating disease symptoms in ayu [17]. Here, we show that harmine treatment significantly dose-dependently suppressed this infection-induced apoptosis compared to controls (Figure 8I,J). To functionally validate PRDX6 as the critical mediator of harmine’s immunomodulatory effects, we performed *PRDX6* knockdown in primary macrophages isolated from ayu (*Plecoglossus altivelis*). Small interfering RNA (siPRDX6) targeting *PRDX6* achieved efficient knockdown at the mRNA and protein levels, with a reduction of approximately 90% compared to control siRNA−transfected cells (Figure 8K,L). In control macrophages (siControl), harmine treatment significantly enhanced bacterial clearance, consistent with our previous observations. However, in *PRDX6*−knockdown macrophages, the baseline bactericidal capacity was markedly enhanced compared to control cells, with a 73.62% reduction in intracellular bacterial burden even in the absence of harmine treatment (Figure 8M). Notably, the ability of harmine to further augment bacterial clearance was significantly attenuated in PRDX6−knockdown cells: while harmine treatment reduced bacterial load by 75.46% in control cells, it achieved only a 2.42% increase in siPRDX6 cells (Figure 8M). This loss of harmine responsiveness in the absence of functional PRDX6 indicates that PRDX6 is essential for harmine’s immunomodulatory activity. These findings collectively demonstrate that PRDX6 is not only a biochemical binding partner of harmine but also a functional target required for its antibacterial-enhancing effects in macrophages.

## 4. Discussion

### 4.1. Dual−Action Pharmacology: Direct Antibacterial and Host-Directed Immunomodulatory Effects

Harmine, a β-carboline alkaloid from *Peganum harmala* seeds, exhibits broad-spectrum antibacterial activity against both Gram−positive and Gram−negative pathogens, including clinically relevant species such as *Staphylococcus aureus* [36], *Acinetobacter baumannii* [16], *Mycobacterium tuberculosis* [37], and *Salmonella enterica* serovar Typhimurium [15] through mechanisms involving biofilm inhibition, membrane disruption, and suppression of the type III secretion system [38]. Despite this established activity, its potential in aquaculture—particularly as an immunomodulator in teleost fish—has remained unexplored, despite the significant threat posed by pathogens such as *P. plecoglossicida* to economically important fish species [39,40]. The present study addresses this gap by demonstrating that harmine exerts potent in vivo antibacterial effects against *P. plecoglossicida* primarily through host-directed immunomodulation rather than through direct bactericidal activity.

A striking discrepancy emerged between harmine’s modest direct bactericidal activity in vitro and its robust efficacy in vivo. Although conventional antibiotics such as ciprofloxacin outperformed harmine in direct killing assays (Figure 1, Figure 2 and Figure 3), harmine showed superior protective effects in infected animals (Figure 4, Figure 5, Figure 6, Figure 7 and Figure 8), suggesting an indirect mechanism involving host immune modulation. Using an integrated target discovery approach combining DARTS and CETSA, we identified peroxiredoxin 6 (PRDX6) as the primary cellular target of harmine. PRDX6 is a multifunctional enzyme central to redox homeostasis and, notably, facilitates the assembly of the TRAF6−ECSIT complex, a critical step in the generation of mitochondrial reactive oxygen species (mROS) during macrophage antimicrobial responses [32]. In teleost fish, where macrophages are a cornerstone of innate immunity due to a less evolved adaptive immune system [41,42], this pathway is of paramount importance. Our results show that harmine binding to PRDX6 liberates TRAF6, enabling its mitochondrial translocation and interaction with ECSIT, thereby amplifying mROS production and enhancing macrophage bactericidal activity. Consistent with this mechanism and with prior studies in avian and murine models [16,43], harmine treatment enhanced macrophage survival, augmented phagocytic and killing capacity, and reduced *P. plecoglossicida*-induced apoptosis.

Critically, PRDX6 also governs inflammatory signaling: its aiPLA_2_ activity is indispensable for NADPH oxidase type 2 (NOX2) activation, generating lysophosphatidic acid to trigger superoxide production, NF-κB nuclear translocation, and NLRP3 inflammasome assembly [44,45]. During bacterial sepsis, lipopolysaccharide (LPS) induces PRDX6 phosphorylation and plasma membrane translocation, amplifying Ca^2+^−independent phospholipase A_2_ (aiPLA_2_) −driven oxidative and inflammatory cascades [46]. Genetic ablation of aiPLA_2_ activity (Prdx6−D140A) or pharmacological inhibition with MJ33 markedly attenuates LPS-induced acute lung injury, vascular inflammation, and mortality, while also modulating cell cycle progression [47,48]. These convergent lines of evidence position PRDX6 not merely as a redox sentinel but as a master regulator of infection-associated inflammation, underscoring its strong potential as a host-directed therapeutic target for sepsis and related hyperinflammatory conditions. Therefore, in the future, we will continue to explore the mechanism of harmine from the perspective of metabolic and immune crosstalk.

Collectively, these findings indicate that harmine achieves its in vivo antibacterial efficacy by reprogramming the host immune response—specifically through activation of the PRDX6−TRAF6−ECSIT−mROS axis—rather than through direct pathogen killing. This host-directed mechanism is especially relevant in aquaculture, where teleost fish rely heavily on innate immune defenses. By leveraging the PRDX6-mROS signaling pathway, harmine effectively compensates for its limited direct antimicrobial activity, resulting in superior in vivo protection. These findings position harmine as a promising immunomodulatory agent for managing bacterial infections in aquaculture, with potential implications for reducing reliance on conventional antibiotics and mitigating antimicrobial resistance.

### 4.2. Limitations and Future Directions

Several limitations of this study must be acknowledged. First, although our harmine dosing regimen (50 and 100 mg/kg intraperitoneal injection) was selected based on preliminary toxicity assays in ayu and resulted in no observable adverse effects, harmine is known to exhibit neurotoxicity in mammals, including central acetylcholinesterase inhibition and convulsant activity at doses approaching 26.9 mg/kg [49]. The narrow therapeutic index of harmine warrants further safety profiling across multiple teleost species and life stages before large-scale aquaculture application can be considered. Second, harmine’s poor aqueous solubility and rapid hepatic metabolism present pharmacokinetic challenges that may limit systemic bioavailability; future formulation strategies, such as nanoparticle encapsulation or the development of soluble derivatives (e.g., harmalacidine hydrochloride), will be essential to achieve consistent therapeutic concentrations.

PRDX6 is a multifunctional enzyme with three biochemically distinct activities—glutathione peroxidase (including unique activity against phospholipid hydroperoxides), aiPLA_2_, and lysophosphatidylcholine acyltransferase (LPCAT)—emerging as a pivotal multifunctional host target [44,45]. This present study focused solely on its glutathione peroxidase activity and signal transduction in monocytes/macrophages in the context of *P. plecoglossicida* infection, as well as its role in stabilizing redox balance to explain its observed antibacterial properties. Further studies should explore its metabolic activity. Although our PRDX6 knockdown experiments confirm the functional role of this target in harmine’s immunomodulatory effects, the long-term consequences of pharmacological PRDX6 modulation in vivo remain unexplored. Studies in human cell lines have shown that PRDX6 knockout can induce G2/M cell cycle arrest and alter lipid signaling pathways, raising concerns about potential off-target effects on teleost growth, embryogenesis, and tissue homeostasis with chronic exposure [50]. Collectively, these limitations underscore the need for comprehensive safety evaluation, formulation optimization, and long-term mechanistic studies to bridge the gap between experimental findings and practical aquaculture applications.

## 5. Conclusions

Harmine exerts potent antibacterial activity against the aquatic pathogen *Pseudomonas plecoglossicida* in vitro by disrupting membrane integrity and inhibiting biofilm formation. In parallel, harmine mitigates infection-induced inflammation and apoptosis in ayu monocytes/macrophages (MO/MΦ), thereby enhancing their phagocytic and bactericidal capacity to promote bacterial clearance. Mechanistically, harmine binds to PRDX6, liberating TRAF6 for mitochondrial translocation and subsequent association with the ECSIT complex. This interaction amplifies mitochondrial reactive oxygen species (mROS) production and maintains redox hemostasis, ultimately potentiating MO/MΦ−mediated bacterial killing.

## Figures and Tables

**Table 1:** Sequence information of primers for qRT-PCR.

Gene	Primer	Sequence	Accession No.
*18S*	18S (+)	GAATGTCTGCCCTATCAACT	FN646593.1
	18S (−)	GATGTGGTAGCCGTTTCT	
*HMGB1*	HMGB1 (+)	GGGGAGACCCCCGGCCCGAC	FR715330.1
	HMGB1 (−)	CGTCCTCTTCCTTCTTCTCG	
*β-actin*	β-actin (+)	TCGTGCGTGACATCAAGGAG	AB020884.1
	β-actin (−)	CGCACTTCATGATGCTGTTG	
*TNFα*	TNFα (+)	ACATGGGAGCTGTGTTCCTC	JP740414.1
	TNFα (−)	GCAAACACACCGAAAAAGGT	
*IL-10*	IL-10 (+)	TGCTGGTGGTGCTGTTTATGTGT	JP758157
	IL-10 (−)	AAGGAGCAGCAGCGGTCAGAA	
*IL-6*	IL-6 (+)	CAATACATGGCCTTGCTTCA	MG264003
	IL-6 (−)	TTGGTCCTCCTTGTTTACCG	
*PRDX6*	PRDX6 (+)	AGATGATAGCCCTCTCTGTG	JP727175.1
	PRDX6 (−)	GGTAGAGGATGGACAACTTC	
*IL-1β*	IL-1β (+)	TACCGGTTGGTACATCAGCA	HF543937.1
	IL-1β (−)	TGACGGTAAAGTTGGTGCAA	
*gyrB*	gyrB (+)	GGTACTCACCTGGTTGGCTT	HE577791.1
	gyrB (−)	GCTTGTCCTTGGTCTGGGAG	

## Data Availability

Data will be made available on request.

## Supplementary Materials

The following supporting information can be downloaded at: https://www.mdpi.com/article/10.3390/antiox15040477/s1, Figure S1: Screening of 30 traditional Chinese medicine (TCM) monomers for antimicrobial activity against *Pseudomonas plecoglossicida*; Figure S2: The cytotoxicity effects of harmine in various normal cells.

## Abbreviations

The following abbreviations are used in this manuscript:

PRDX6	Peroxiredoxin 6
TRAF6	TNF receptor-associated factor 6
ECSIT	ECSIT signaling integrator
BHA	Bacterial hemorrhagic ascites
T3SS	Type III secretion system
TCM	Traditional Chinese medicine
OD	Optical density
CFU	Colony-forming unit
PBS	Phosphate-buffered saline
TSA	Tryptic Soy Agar
MIC	Minimum inhibitory concentration
TSB	Tryptic Soy Broth
CETSA	Cellular thermal shift assay
CCK-8	Cell Counting Kit-8
RT-qPCR	Quantitative real-time PCR analysis
MO/Mφ	Monocytes/macrophages
MFI	Mean fluorescence intensity
AST	Glutathione peroxidase aspartate aminotransferase
ALT	Alanine aminotransferase
ALP	Alkaline phosphatase
SOD	Superoxide dismutase
CAT	Catalase
MDA	Malondialdehyde
DARTS	Drug Affinity Responsive Target Stability
LC-MS/MS	Liquid chromatography-tandem mass spectrometry
Cip	Ciprofloxacin
NF-κB	Nuclear factor kappa B
TNF-α	Tumor necrosis factor
IL-6	Interleukin-6
HMGB1	High Mobility Group Box 1
ROS	Reactive oxygen species
mROS	Mitochondrial reactive oxygen species
