# Supplementary material for: Harmine Targets Peroxiredoxin 6 to Enhance Macrophage Immunity Against Pseudomonas plecoglossicida in Ayu (Plecoglossus altivelis)
Source: Antioxidants (Basel). 2026 Apr 11;15(4):477. doi: 10.3390/antiox15040477 (PMC13113785; doi:10.3390/antiox15040477)
Supplement: Supplementary file 1 [file antioxidants-15-00477-s001.zip › antioxidants-4192392-supplementary.pdf]

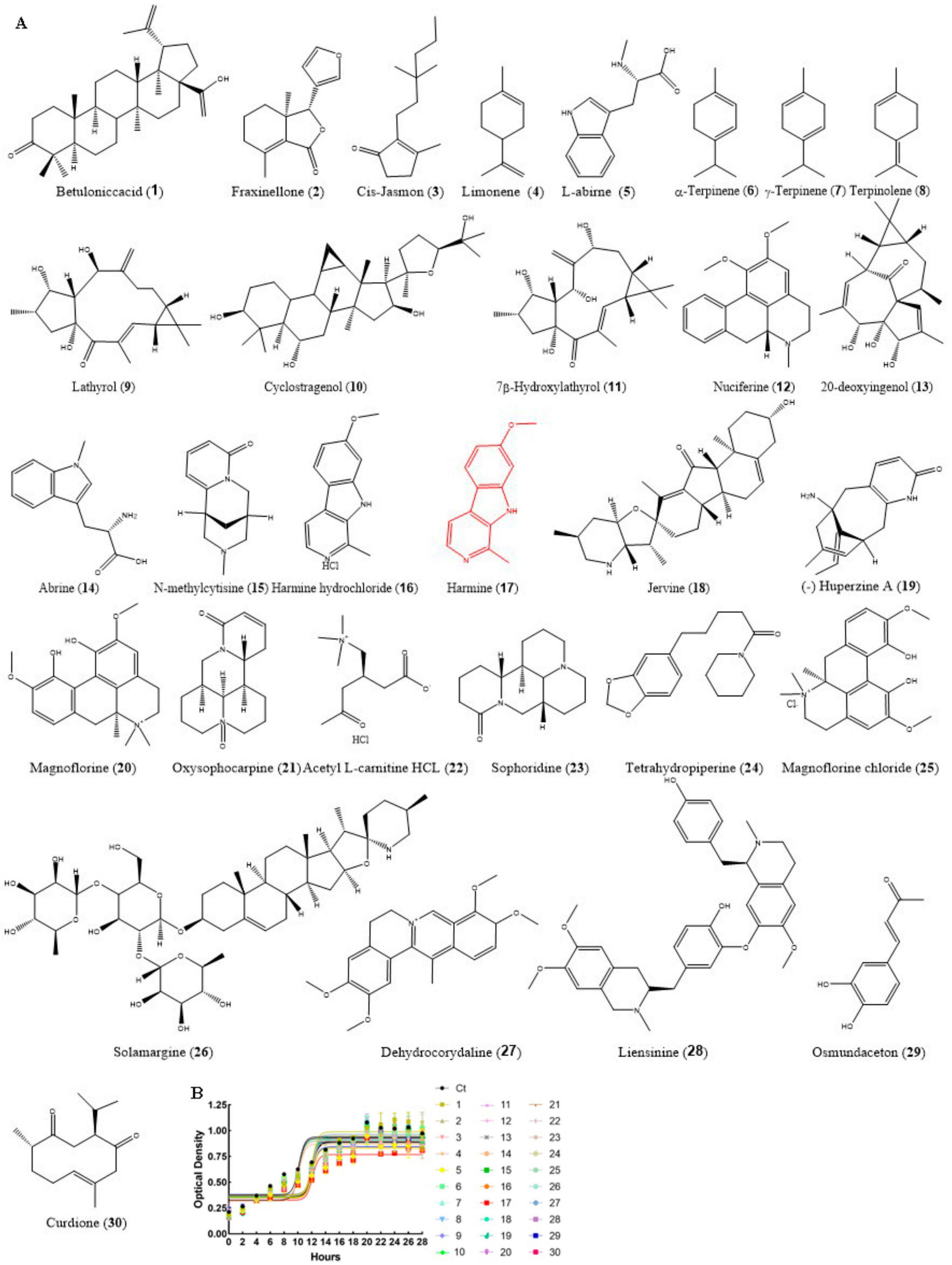

Figure S1. Screening of 30 Traditional Chinese Medicine (TCM) monomers for antimicrobial activity against *Pseudomonas plecoglossicida*. (A) Chemical structures of the 30 tested TCM monomers. (B) Growth curves of *P. plecoglossicida* in the presence of each TCM monomer at 25 μM (compared to untreated control).

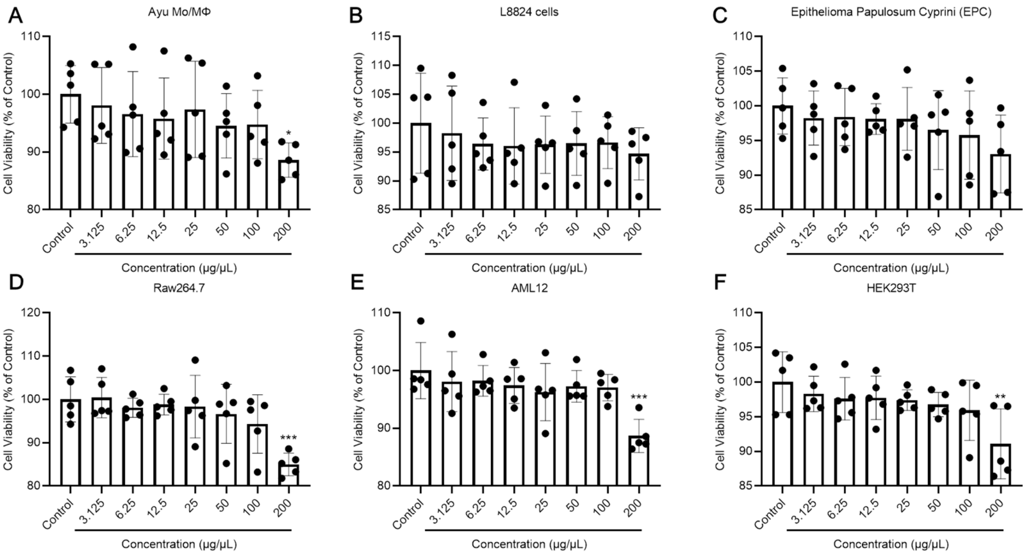

Figure S2. The cytotoxicity effects of harmine in various normal cells. (A) Ayu MO/Mϕ, (B) L8824 cells, (C) EPC cells, (D) Raw 264.7 cells, (E) AML12 and (F) HEK293T cells. $^{*}p < 0.05$, $^{**}p < 0.01$ and $^{***}p < 0.0001$ compared to the control group.
